# Multilevel Determinants of Engagement in Lifelong Learning in Orthodontics From Formal to AI-Supported Approaches: Development of a Conceptual Framework Through a Qualitative Study

**DOI:** 10.2196/85634

**Published:** 2026-05-14

**Authors:** Theerasak Nakornnoi, Jitpitchaya Wimonchaijit, Rochaya Chintavalakorn, Kawin Sipiyaruk

**Affiliations:** 1 Department of Orthodontics, Faculty of Dentistry, Mahidol University, 6 Yothi Road, Ratchathewi, Bangkok, 10400, Thailand, 66 22007813

**Keywords:** continuing education, dental education, lifelong learning, orthodontics, qualitative research

## Abstract

**Background:**

Lifelong learning (LLL) is increasingly important for health care professionals, particularly within the field of orthodontics, driven by emerging technologies, updated treatment techniques, and rising patient expectations. To maintain competence in current practice, orthodontists are expected to engage in continuous professional development throughout their careers. While the value of LLL is widely acknowledged, engagement levels among orthodontic professionals can vary due to a number of factors.

**Objective:**

This study aims to investigate how orthodontic professionals perceive and engage with LLL by examining their learning strategies, along with the barriers and enablers. In addition, this study integrates these determinants of professional development to construct a conceptual framework to explain LLL engagement among orthodontic professionals.

**Methods:**

A qualitative study using semistructured interviews with a topic guide was conducted among 16 orthodontic professionals who were recruited using purposive sampling to ensure diversity in settings and experience levels. Interviews were audio recorded and transcribed verbatim until data saturation was achieved. Data were analyzed using framework analysis informed by the theory of planned behavior. To ensure analytic rigor and credibility, an iterative coding process was performed independently by 2 researchers, with any discrepancies resolved through consensus among the research team.

**Results:**

All participants believed that LLL was important for clinical improvement and personal fulfillment. However, they reported several challenges, including time constraints, financial restrictions, and limited access to learning resources. Social influences, institutional contexts, technological advancements, and patient expectations were considered key determinants in shaping LLL engagement. The results also revealed a pedagogical shift in LLL activities from formal short course training to the use of generative artificial intelligence for self-directed learning. A conceptual framework illustrating key determinants of LLL engagement was developed.

**Conclusions:**

LLL engagement was perceived as essential for clinical competence and personal development, which could be influenced by individual, social, institutional, and external factors. The conceptual framework developed in this study demonstrates the key determinants of LLL in orthodontic practice and suggests that multilevel strategies are required to sustain long-term engagement.

## Introduction

Lifelong learning (LLL) appears to be an essential skill for health care professionals. This competency could be considered a required characteristic in the field of orthodontics, as the recent environment of orthodontic practice has become increasingly unpredictable and complex [1]. There is an increasing use of technologies, ranging from advanced imaging techniques to digital treatment planning and customized appliances, resulting in the improvement of treatment outcomes when compared with conventional approaches [2-5]. These technologies are perceived as significant by orthodontic professionals in clinical workflow [6-9]. Furthermore, the demographics of orthodontic patients underscore the importance of adopting patient-centric approaches and interdisciplinary collaboration to address their diverse needs and expectations of patients [10]. In addition, health care professionals with a strong commitment to LLL can perform better in navigating and managing clinical challenges [11]. Consequently, LLL has become fundamental to maintaining professional standards in modern orthodontics.

While a number of learning methods have been used to update knowledge and skills for continuous improvement in patient care [12], evidence demonstrates that orthodontic professionals may have unique barriers to engaging in LLL activities. Interestingly, orthodontic professionals appear to exhibit lower levels of engagement in continuing education activities compared to other dental specialties [13]. Common challenges appear to be busy schedules of clinical work, which make it difficult to balance professional and personal tasks [14,15]. The high cost of courses and training seems to be another significant barrier, in particular for those with financial constraints [16,17]. Furthermore, unequal access to technology can limit learning opportunities in the digital age [17-19], while the large amount of online information can leave professionals feeling overwhelmed and struggling to identify trustworthy sources [20]. These barriers can negatively impact professional development, highlighting the need to understand LLL behaviors and attitudes within orthodontic professionals to better support their ongoing development.

The LLL engagement in orthodontic education can be considered complicated due to a number of unique factors. Evidence reveals a generation gap, where younger and older orthodontists perceive barriers toward evidence-based practice differently [21]. This finding is consistent with broader trends observed in medical education, where residents frequently get new information by self-directed learning from internet searches, while experienced clinicians tend to draw upon their past experiences [22]. Furthermore, a study confirms that natural cognitive changes associated with aging can influence learning preferences over a long career [23]. These approaches create an educational landscape, highlighting the need to move beyond a one-size-fits-all model and investigate how orthodontic professionals actually perceive and engage with LLL.

Although available evidence has explored LLL in a variety of health care disciplines, little research has been performed on the unique learning behaviors of orthodontic professionals. To enhance the quality of orthodontic care, it is necessary to understand how they engage in LLL activities. Therefore, this study aimed to explore how orthodontic professionals perceive LLL and to investigate its relevant determinants. By identifying these factors, this study seeks to provide insights into supporting dental educators to effectively develop more personalized training approaches, ensuring safe and high-quality patient care within orthodontic practice. To achieve this aim, the following research questions were formulated**:**

1. How do orthodontic professionals currently engage in LLL activities, ranging from formal education to artificial intelligence–supported approaches?
2. How do orthodontic professionals perceive the value of LLL in their practice?
3. What are the key enablers and barriers affecting their engagement in LLL activities?
4. How can these determinants be integrated into a conceptual framework to explain LLL engagement in orthodontics?

## Methods

### Theoretical Framework

**Figure 1.**
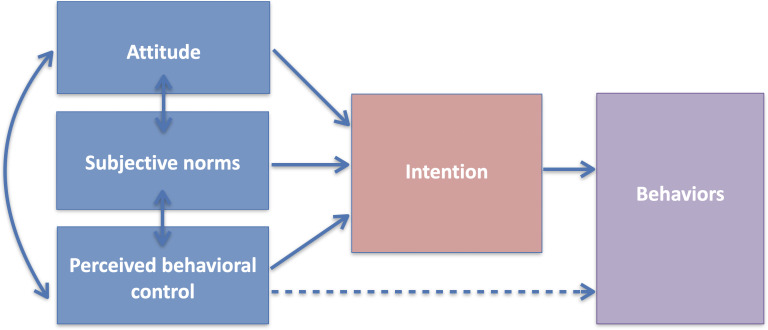
Theory of planned behavior.

The theory of planned behavior (TPB) was selected as the underpinning theoretical framework for this study (Figure 1), as LLL can be considered a complex behavior rather than a purely cognitive phenomenon. According to TPB, behavioral intentions are shaped by 3 core constructs, which are attitudes, subjective norms, and perceived behavioral control [24]. According to this theory, individuals are more likely to engage in a behavior when they hold positive attitudes, perceive support from influential others, and feel confident in their ability to perform it.

Existing research evidence in health care education has successfully applied TPB to explain engagement in professional activities. For instance, attitude and perceived behavioral control were found to be significant predictors of intentions to adopt technology-enhanced learning in continuing medical education among general practitioners [25]. In addition, there is scientific evidence demonstrating that stronger intentions to engage in targeted professional practices are associated with more positive attitudes, supportive social norms, and greater perceived control [26]. Therefore, TPB was considered a suitable framework to serve as a guide without rigidly constraining the analysis in this study. In other words, while the theory provided an initial structure for the analytical framework, the qualitative nature of this research allowed the researchers to uncover additional context-specific determinants from the data, further enriching the understanding of engagement in LLL activities.

### Research Design

This study used a qualitative research design using semistructured interviews to gather in-depth information from orthodontic professionals. The qualitative approach was chosen, as it could effectively capture the personal perspectives and insights of orthodontists regarding LLL that would be difficult to quantify through quantitative research [27]. As LLL can be influenced by individual attitudes, professional community and culture, personal challenges, and other contextual factors, qualitative research should provide the most appropriate way for exploration. With multilevel determinants and multifaceted factors, this study adopted a hybrid deductive-inductive approach. This qualitative study was conducted within an interpretivist paradigm and used a framework analysis approach informed by TPB. The deductive component used TPB to structure the inquiry around established psychological constructs, while the inductive component allowed for the emergence of novel themes and context-specific insights directly from the participants’ experiences.

### Research Participants

The research population included orthodontic professionals who were either enrolled in or had graduated from postgraduate programs in orthodontics at Mahidol University. However, they were excluded if they were no longer active in orthodontic practice. A purposive sampling technique was used to select participants for the interviews to ensure that diverse and information-rich participants were selected to provide their insights [28]. The sampling criteria were considered based on sex (female, male), age (26‐75 years), years of orthodontic experience (categorized as 1‐3 years, 3-10 years, 11‐20 years, and over 20 years), and practice setting (including postgraduate residents, university instructors, private practitioners, and private practice owners). Potential participants were approached via professional networks. No individuals who were approached declined participation, and no interviews were withdrawn or terminated prematurely. The final sample size comprised 16 participants, determined by data saturation [29], where there were no new themes or subthemes that emerged from subsequent interviews.

### Data Collection Procedures and Topic Guide Design

Semistructured interviews with a topic guide were performed to collect qualitative data from research participants. The topic guide was developed based on the TPB concept [24]. Accordingly, the topic guide covered 5 main areas, which were: “Professional background,” “Perceived importance of LLL (Attitudes),” “Professional community and social expectations (Subjective norms),” “Perceived control over and barriers to learning opportunities (Perceived behavioral control),” “Behavioral intentions,” and “Actual learning practices.” The interviews were conducted in a private room, where only the participant and the assigned researcher (JW) were present, to ensure privacy and confidentiality. All interviews were conducted in Thai to preserve the authenticity of perceptions and expressions captured from research participants. Each interview lasted approximately 40 to 50 minutes and was audio-recorded after obtaining informed consent from each participant. Field notes and analytic memos were documented during and after interviews. The recordings were then transcribed verbatim using a confidential transcription service. All transcriptions were then anonymized before the analysis process.

### Data Analysis

Data analysis was conducted using framework analysis [30], where a preliminary thematic framework was based on the TPB model [24]. This data analytic approach allowed researchers to integrate new themes and subthemes that emerged from the interview data into the original framework. All interviews were conducted in Thai, but they were translated into English using a double-translation method to confirm the accuracy of the translation. Firstly, the interview transcripts were translated from Thai to English by an external translator. Another independent translator then back-translated them into Thai. The original and back-translated versions were then compared by the research team to evaluate whether the English-translated quotes accurately reflected the original meaning. Any discrepancies in the translations of the transcripts were revised to ensure accuracy before analysis. Therefore, the data analysis was performed on the translated English transcripts.

The verified English transcripts were imported into *Delve,* a qualitative data analysis tool, to facilitate systematic coding and transparent management. To enhance the trustworthiness of the analysis, all procedures were performed and documented with transparency, allowing them to be reviewed and validated. The coding process followed an iterative procedure to ensure the analytic rigor of the study. First, an initial coding framework was independently developed based on the core TPB constructs (attitudes, subjective norms, perceived behavioral control, intention, and behavior) by 2 researchers (JW and KS). After the initial round of coding, the research team reviewed and compared their decisions, during which any identified discrepancies in the development of themes and subthemes were discussed until a consensus was achieved to ensure consistency and credibility in the analysis. The revised coding framework was then indexed to the entire dataset by JW. During this stage, the coding process was regularly reviewed by KS and TN to ensure consistency. Regular discussions were arranged to maintain reflexivity throughout the analysis process, with identified codes being adjusted to ensure conceptual clarity and coherence across all transcripts. Finally, to ensure confirmability and accuracy, the themes and the conceptual framework were reviewed and validated by an external expert in dental education.

### Ethical Considerations

This study protocol was reviewed and approved by the Institutional Review Board of the Faculty of Dentistry and the Faculty of Pharmacy, Mahidol University (certificate of approval number MU-DT/PY-IRB 2024/040.1207). All methods were performed in accordance with the relevant guidelines and regulations. Informed consent was obtained from all participants. To protect confidentiality, all data were anonymized, and no personally identifiable information was available during analysis. No compensation was provided to participants.

## Results

### Research Participants

The research participants included 16 orthodontic professionals, comprising 5 postgraduate residents, 4 university instructors, 5 private practitioners, and 2 dental practice owners. They consisted of 6 male and 10 female participants. The age range of the participants was 29 to 75 years. Their information is presented in Table 1.

**Table 1.** Participants included in this study.

Participant	Sex	Age range (years)	Practice setting	Years of orthodontic experience
1	Female	26‐30	Postgraduate resident	1‐3
2	Male	26‐30	Postgraduate resident	1‐3
3	Female	26‐30	Postgraduate resident	1‐3
4	Female	31‐35	Postgraduate resident	1‐3
5	Male	31‐35	Postgraduate resident	1‐3
6	Female	31‐35	University instructor	3‐10
7	Female	31‐35	University instructor	3‐10
8	Male	41‐45	University instructor	11‐20
9	Female	46‐50	University instructor	11‐20
10	Female	31‐35	Private practitioner	3‐10
11	Female	36‐40	Private practitioner	3‐10
12	Female	61‐65	Private practitioner	Over 20
13	Male	61‐65	Private practitioner	Over 20
14	Male	71‐75	Private practitioner	Over 20
15	Female	36‐40	Private practice owner	3‐10
16	Male	41‐45	Private practice owner	11‐20

### Themes and Subthemes Emerged from the Framework Analysis

Following the framework analysis, 7 themes were identified: (1) attitude: impact of LLL on professional practice, (2) subjective norms: social influence on LLL in orthodontics, (3) perceived behavioral control, (4) intention to engage in LLL, (5) learning behaviors and methods, (6) institutional context: organizational and workplace environment, and (7) external drivers of LLL. Although these themes are interconnected, each represents a distinct dimension of how orthodontists experience and engage in LLL, from personal attitudes and social influences to institutional contexts and external drivers, such as technological changes and patient expectations.

#### Theme 1: Impact of LLL on Professional Practice (Attitude)

Participants consistently considered LLL to be very important for their clinical practice and personal development. They perceived LLL as a mechanism to enhance patient care, increase treatment efficiency, and ensure their knowledge remains up-to-date. Learning was also linked to personal fulfillment.

##### Improvement in Clinical Practice

Most participants emphasized that LLL was essential for adapting to a field where knowledge and technology evolve continuously. With current technologies, orthodontists can simplify complex procedures and improve clinical efficiency. As a participant stated, “[It] helps us do our work more easily [and] reduces chair time and patient visits” [Participant 16]. Participants also noted that failing to update knowledge on evolving technologies can hinder the ability to optimize their clinical outcomes. For instance, a participant explained, “We need to adapt and apply knowledge ... like with clear aligners ... now the lab design has improved, ... If we do not update our knowledge, we would not know how to simplify things” [Participant 9].

##### Personal Fulfillment

Participants considered LLL as a way to fulfill intrinsic motivation, describing it as a mechanism to grow professionally and maintain personal relevance in a dynamic field. Learning was often associated with self-satisfaction, intellectual stimulation, and the desire to become better each day, not just for their patients, but for their own sense of purpose. For instance, a participant expressed a personal passion for LLL, stating, “I enjoy learning ... I feel motivated to keep learning and expanding my knowledge” [Participant 11]. Furthermore, participants believed that LLL provided intellectual stimulation, particularly for those who sought to validate new trends or treatment techniques through research rather than accepting them blindly. As 1 participant explained, “[I want] to see if it really works. ... I enjoy doing research and learning to verify whether claims are true or not” [Participant 7].

### Theme 2: Social Influence on LLL in Orthodontics (Subjective Norms)

Participants reported that LLL could be influenced by perceived subjective norms, particularly from peers and mentors, whose behaviors and expectations affect participants’ motivation to engage in continuous professional development.

#### Peer Influence

Participants highlighted that peers played a role in motivating them to explore new knowledge and concepts, and that social comparison often reinforced their intention to remain up-to-date. Attending academic conferences with friends made the experience “more enjoyable” [Participant 1]. In addition, social comparison could reinforce the intention to remain up-to-date, as 1 orthodontic resident stated that seeing peers advance created a competitive motivation: “If my friends are attending something, I want to go and learn with them. If my friends know more, I sometimes want to know too” [Participant 4].

#### Mentor Influence

Mentors and senior colleagues were perceived as role models in guiding the direction of learning, especially during the early years. Their recommendations could shape learning priorities and introduce new areas of interest or clinical trends. For instance, 1 resident highlighted how a mentor influenced her to engage in LLL activities, noting that her professor “advised me to regularly attend conferences ... [and] to present my works at least once a year” [Participant 1].

### Theme 3: Perceived Behavioral Control

Participants identified multiple factors that influenced their ability to engage in LLL, which they described in terms of both challenges that created barriers and enablers that supported greater access and engagement.

#### Challenges to Participating in LLL Activities

##### Time Restrictions and Workloads

Time constraints and heavy workloads were frequently cited as barriers by participants, particularly for those in private practice or busy hospitals. Participants described being overwhelmed by clinical hours and administrative duties, which left little opportunity for professional development. For example, 1 participant noted that, while they understood concepts like 3D printing, they lacked the “time to learn the manual process [and] study the back-end details” [Participant 16]. Similarly, others reported reducing their attendance at orthodontic conferences and events because of “clinical duties and family responsibilities” [Participant 8], even though they considered these events very helpful.

##### Financial Constraints

High registration fees for courses and conferences were perceived as a major barrier. Additional expenses for travel and accommodation, especially for events held abroad or outside participants’ towns, further discouraged attendance. Participants often weighed costs against potential benefits, noting that “if I feel a course is too costly for what I will gain, that becomes a barrier” [Participant 7]. This challenge was more emphasized for orthodontists in provincial or rural areas. For them, traveling to major cities meant not only paying for transport and accommodation but also losing income from their routine clinical work. As a participant explained, “That means closing my clinic for a few days, so it is not just the ticket, but it is the income I lose” [Participant 15].

##### Limited Access to Learning Resources

Limited access to high-quality academic resources was another challenge, particularly for those in private practice without institutional subscriptions. Participants mentioned that accessing leading journals was difficult because “some journals still require payment” [Participant 16].

##### Resistance to Change

This barrier was also observed, often stemming from a reliance on existing techniques or a lack of confidence in adopting new technologies. Although participants did not share this view themselves, they observed that some colleagues might avoid innovation because “they feel they cannot keep up” [Participant 15]. Furthermore, if practitioners feel they can “manage all cases effectively using their existing methods” [Participant 12], they may perceive no need for further learning.

### Enablers to Engage in LLL Activities

Beyond the challenges, participants highlighted several factors that made it easier for them to stay engaged in LLL activities. One of the most frequently mentioned enablers was the growing accessibility of digital tools and online platforms. Resources such as Google, YouTube, and online conferences were described as convenient ways to check ideas, review concepts, or explore unfamiliar topics. In addition, modern tools like ChatGPT were noted for making it “much easier to find information in real time,” albeit with a caution that users “still have to be careful” regarding accuracy [Participant 8]. Similarly, the transition to digital platforms has reduced the need to rely solely on physical copies or in-person events, offering a “much greater variety of resources available today” [Participant 9].

### Theme 4: Intention to Engage in LLL Activities

This theme reflects a forward-looking commitment to professional growth, which was described in different ways. While participants were keen to explore new technologies like AI, they maintained a critical perspective, noting that currently, “marketing tends to come out before the science” [Participant 8]. Consequently, they expressed a strong intention to verify “reliable data” [Participant 8] and investigate “if it [AI] can actually help us” [Participant 9] before adoption. Furthermore, specific learning goals often drove this engagement. As 1 participant stated, “There are still areas I would like to explore ... improving my digital workflow ... [and] understanding how to use AI in clinical decision-making” [Participant 12].

### Theme 5: Learning Behaviors and Methods

This theme demonstrates how participants engage in LLL activities through formal and informal methods. Although all participants had undergone structured orthodontic training, such as residency or postgraduate programs, they used a variety of strategies, ranging from formal short-course training to the use of generative AI for LLL engagement. This reflects a shifting paradigm in orthodontic education, where convenience, interactivity, cost, and trust influence individual choices.

#### Formal Short-Course Training

Several participants reported that continuing education provided opportunities to update knowledge and skills. Participants demonstrated a strong preference for in-person sessions over online formats, as these activities allowed for more engagement and real-time responses to their questions. These courses reflect a traditional educational model that is still highly valued for hands-on guidance. As a participant noted regarding their training in clear aligners, “It is easier to have somebody teach you rather than learning by yourself” [Participant 9], highlighting a preference for direct instruction over self-study for complex clinical skills.

#### Scientific Evidence

Scientific evidence from trusted sources, such as academic articles, was considered the most convenient and reliable method reported by most participants. Online access to these articles provided flexibility, allowing them to validate clinical knowledge at any time. One participant explained that this is their initial step: “When I want to know something new, first I read papers from top tier journals ... [and] try to find evidence to support” [Participant 5]. However, some participants noted that this approach was time-consuming. Another participant remarked that while they seek evidence-based support, “working in a full-time private practice, it takes me too long to understand all of this” [Participant 15].

#### Online Learning

Online learning sessions, recorded lectures, and on-demand webinars were popular among participants because they offered flexibility, cost-effectiveness, and the elimination of travel burdens. These digital formats represented a shift toward learner-controlled approaches, allowing busy clinicians to access content from reliable sources at their convenience. As a participant explained regarding the benefits of online learning materials like webinars, “I can replay it anytime, and when I forget something, these recordings help me recall the information easily” [Participant 12]. However, participants acknowledged that online learning could make them feel less engaged, as this approach lacked the interactivity of in-person education. In addition, while online platforms were convenient for studying theoretical concepts, participants found them insufficient for complex clinical tasks. One participant noted that despite attending many web-based sessions, “online learning alone is not enough ... I still need real cases and experienced professors to guide me” [Participant 3], emphasizing that expert mentorship remains essential for consolidating practical skills.

#### Conference-Based Learning

Conferences were considered by many participants to support professional learning. They were described as high-value events, particularly when featuring well-known speakers, real case presentations, or hands-on workshops. Compared to online formats, conferences offered networking activities, and participants were able to focus on learning while being away from daily clinical demands. Although expense and time were acknowledged as constraints, attendance was considered worthwhile if the content aligned with their interests and featured authoritative experts. As a participant emphasized, “a good speaker makes you really learn ... [and] if that person has solid foundational knowledge, then it is worth” [Participant 9], noting that the decision to attend often depended on the speaker’s reputation regardless of the location.

#### Peer Learning

Participants noted that peer discussion played an important role in everyday learning. This method was valued because it was practical, trustworthy, and highly contextualized, particularly when advice came from experienced colleagues. For instance, these platforms provide a supportive setting to “share clinical experiences and review real-world cases from senior colleagues” [Participant 8]. Furthermore, the immediacy of digital communication allows for rapid problem-solving; as 1 participant described regarding a messenger group, colleagues can “post a photo with a question, and others can quickly provid responses” [Participant 9]. This highlights how learning has extended beyond classrooms and conferences, moving from formal toward more informal activities and community-driven exchanges through professional communication groups.

#### Generative AI

Generative AI tools were increasingly considered emerging supplementary resources, valued for their speed and accessibility compared to formal courses. These tools facilitate on-demand exploration, allowing clinicians to “learn at [their] convenience” [Participant 1] and obtain immediate overviews. However, trust in these tools was tempered by caution regarding accuracy, particularly for clinical details. Participants emphasized that while generative AI is useful for quick inquiries, it is “often not right if you ask for references” [Participant 1]. Therefore, it served as a preliminary tool rather than a definitive source, with participants stressing the need to “double check the information with other sources” [Participant 9] to ensure validity. Consequently, generative AI was integrated not as a replacement for traditional approaches but as a supplementary aid, signaling a potential paradigm shift in orthodontic education toward AI-driven support integrated into everyday learning.

### Theme 6: Organizational and Workplace Environment (Institutional Context)

In addition to personal-level barriers and enablers (addressed in Theme 3), this theme explores factors at the organizational level that shaped how orthodontists were engaged in LLL activities, including institutional policies and workplace culture.

#### Institutional Policies as Barriers

For participants in public hospitals and large organizations, operational requirements often outweighed personal motivation. Significant structural barriers, particularly strict leave policies and staff shortages, frequently prevented attendance at training events, even when participants possessed a strong willingness to learn. One participant described this conflict, citing instances where they could not attend “because there were not enough staff [or they] had already used up [their] leave” [Participant 7], noting that such logistical hurdles were a defining and persistent challenge during their tenure in the hospital system.

#### Supportive Workplace Culture

Academic institutions and teaching hospitals fostered an environment where LLL was a professional expectation. This culture was reinforced by support such as funding and flexible scheduling. One participant highlighted how this comprehensive support directly influenced their decision to engage in learning activities: “The university covered my registration fee, hotel and flight tickets to attend the meeting, so I thought ... why not go?” [Participant 2]. Similarly, the specific role of an educator created an intrinsic motivation to maintain high educational standards. As another participant explained, “Being part of an academic institution pushes you to stay sharp. When you are expected to teach or supervise, you cannot let your knowledge get stale” [Participant 6].

### Theme 7: External Drivers of LLL

This theme examines the external forces that influence LLL engagement, extending beyond personal motivation and institutional context. Participants identified 2 primary external drivers: technological advancements in orthodontics and patient expectations.

#### Technological Advancements in Orthodontics

Participants considered advances in orthodontic technology as an external influence on their commitment to LLL. The increasing integration of digital innovations, ranging from in-house aligner production and 3D scanning to AI-assisted treatment planning, has transformed routine clinical workflows, necessitating continuous skill acquisition to maintain competency. One participant highlighted clear aligner therapy as a significant market force that compelled them to adapt, stating: “The most recent disruption would be the clear aligner ... The new materials have reached a good level. … I have to learn it” [Participant 8]. This reflection underscores the professional imperative to master emerging technologies once they reach a level of maturity that impacts standard practice.

#### Patient Expectations

Patient expectations significantly shaped LLL engagement among orthodontic professionals. As patients increasingly accessed dental information online, they often asked about new treatment techniques, prompting orthodontists to recognize the need to maintain professional authority. As 1 participant noted, “Patients are more informed ... If we do not know, how can we explain or treat?” [Participant 12].

Additionally, patient demand often dictated the adoption of new techniques. One participant admitted that specific inquiries drove their skill acquisition, stating: “Patients requested self-ligating brackets, ... [and] their requests pushed me to learn more. … The same happened with aligners” [Participant 16].

### A Conceptual Framework of Key Determinants of LLL Engagement Among Orthodontic Professionals

**Figure 2.**
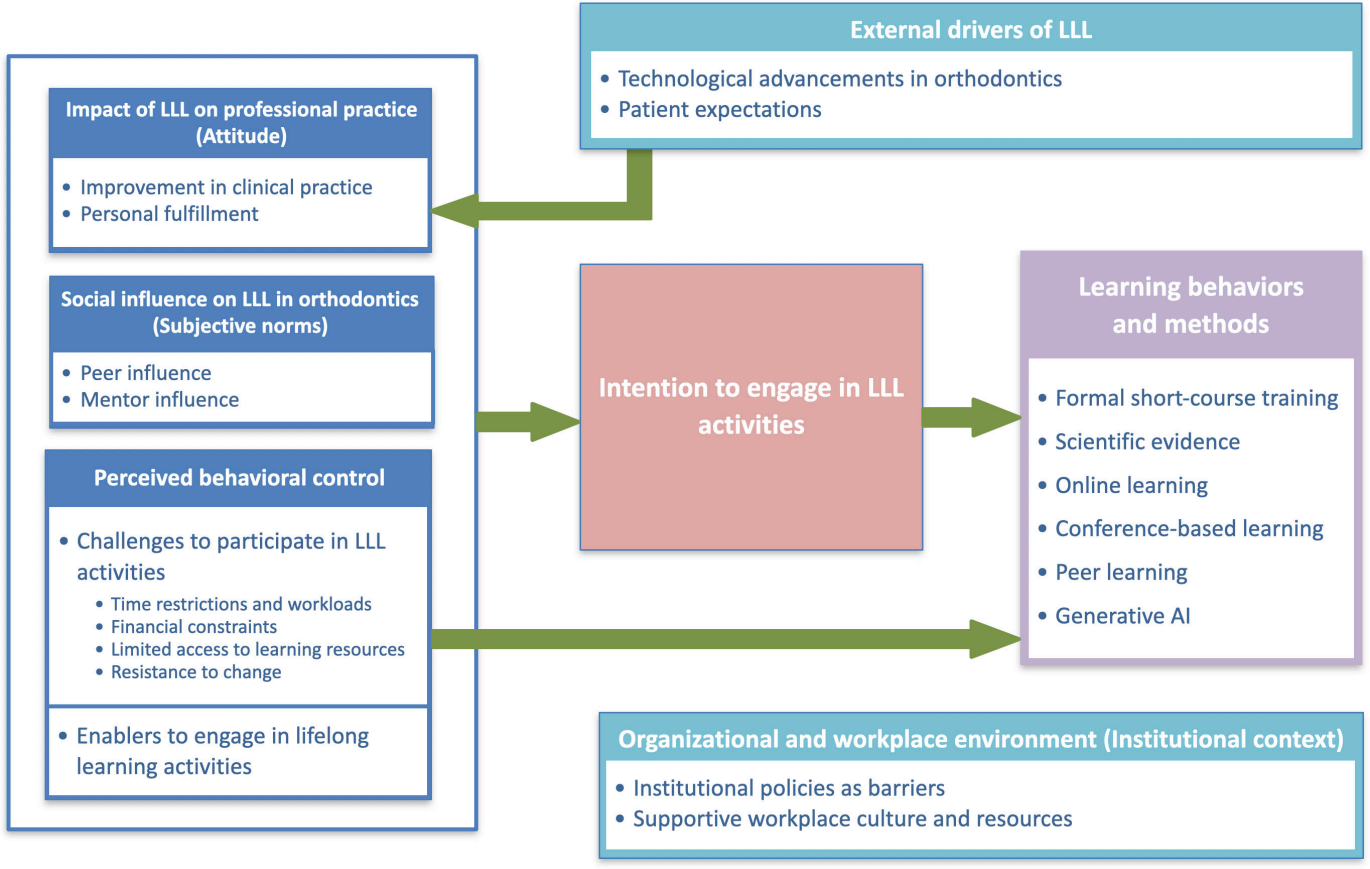
Conceptual framework of key determinants of lifelong learning engagement among orthodontic professionals. AI: artificial intelligence; LLL: lifelong learning.

A conceptual framework was developed through framework analysis of qualitative data retrieved from semistructured interviews (Figure 2), demonstrating the interrelationships among the thematic elements. The framework provides an understanding of how key determinants could shape LLL engagement among orthodontic professionals through TPB, shifting from formal short-course training to more flexible approaches such as the use of generative AI. The framework highlights that the engagement of orthodontic professionals in LLL can be sustained not only by personal motivation, social influence, and perceived behavioral control but also by institutional factors and external pressures. This suggests that fostering LLL engagement among orthodontic professionals requires multilevel strategies, ranging from cultivating positive individual attitudes and creating supportive peer and mentor networks to leveraging external drivers to reinforce the value of continuous learning.

## Discussion

### Key Findings and Conceptual Framework

This study aimed to explore how orthodontic professionals perceived and engaged in LLL, identify the strategies they adopt, and examine the determinants influencing such engagement using a TPB-informed framework. The findings revealed that although LLL was widely perceived by orthodontic professionals as essential for clinical efficiency and personal fulfillment, actual engagement (learning behaviors and methods) can be shaped by the interaction between individual attitudes, social influence (mentors and peers), perceived behavioral control, and institutional contexts. These factors were further driven by external pressures, specifically technological disruptions and patient expectations. These determinants were integrated into the conceptual framework, informing how orthodontic professionals consider potential enablers and barriers to make strategic decisions regarding their engagement in LLL.

### Professional Impact and Personal Fulfillment

The important theme that emerged was the impact of LLL on professional practice, which aligned with the “Attitude” construct of the TPB model [24]. LLL is a necessary skill for ensuring patient safety and maintaining a high level of orthodontic competence, which is consistent with earlier research demonstrating that it can improve treatment outcomes and strengthen evidence-based practice [10,31]. In addition to the professional aspect, this research highlights that LLL engagement could be driven by personal fulfillment. This reflects intrinsic motivation, where they are satisfied by acquiring new skills or staying updated with rapid technology advancements, as a key driver of adult learning engagement [32]. They perceive LLL as a fundamental part of being a professional rather than just a mandatory requirement [33]. Therefore, both intrinsic and extrinsic motivations are required to engage orthodontists in LLL activities.

#### Barriers and Enablers to LLL Engagement

When considering “Perceived behavioral control,” common barriers to LLL engagement included time restrictions, heavy workloads, financial concerns, and resistance to change, consistent with other studies [13-15,34]. Work-family conflicts and institutional barriers can also be cited as major barriers to continued professional development [35]. At the same time, new learning-aid technologies such as asynchronous learning platforms, webinars, and AI-driven tools have been acknowledged as important facilitators, especially for younger practitioners [36]. With immediate feedback offered by these educational technologies, orthodontic professionals can engage in self-directed learning as a part of their LLL activities [37]. Consequently, despite a number of challenges, the implementation of technologies can facilitate orthodontists to engage in LLL activities.

### Mentors and Peers as Social Influence

“Social influence on LLL in orthodontics,” corresponding to the “Subjective norms” construct in the TPB model, emerges as another key theme in this research. Mentors and peers can significantly impact LLL engagement, providing support for LLL. Mentorship can offer opportunities for knowledge sharing and skill development to support professional growth [38]. Peers can also have a positive impact on LLL engagement through informal encouragement, such as inviting colleagues to conferences, sharing new tools and techniques, and discussing orthodontic cases. Collaborative peer learning can positively influence academic growth and offer emotional support, leading to the enhancement of learning engagement [39]. These informal situations can offer learning opportunities, where social influence can encourage orthodontists to engage in LLL activities.

#### Institutional Contexts and External Drivers

In addition to the constructs available in the TPB model, organizational contexts and external drivers can impact LLL engagement among orthodontic professionals. Academic or hospital-based practitioners could benefit from educational time allowances and formal faculty development programs, where a learning culture can be cultivated [40]. Meanwhile, private practitioners are usually required to finance their learning activities on their own, resulting in difficulties in balancing educational pursuits with business needs [15]. Furthermore, the increasing number of patients who expect advanced technologies can drive practitioners to continue their education [10,41]. These contextual and external influences demonstrate that engagement in LLL can extend beyond the constructs of the TPB model.

### Generational Shifts in Learning Preferences

The intention to engage in LLL is reflected in a wide range of learning methods, revealing a significant generational transition. Rather than formal training, younger professionals tend to prefer more flexible learning approaches [42-44]. Based on the interview data, they were likely to engage in LLL activities using online learning communities and generative AI. These results align with other studies, showing an increasing trend of digital literacy in health care education [45,46]. In addition, younger orthodontic professionals tend to use generative AI in supporting their learning and decision-making in complex tasks [47]. These strategies highlight a paradigm shift in LLL engagement, where younger generations of orthodontic professionals tend to prefer more flexible approaches in supporting their professional and career growth.

### Implications for Orthodontic Education

Another important consideration is to use the conceptual framework to inform the implications of these findings for enhancing LLL communities among orthodontic professionals. Institutions and professional bodies should consider flexible learning communities or funding for continuing education. Simulations and serious games can also be used as asynchronous interactive learning platforms to support LLL engagement for dental professionals, including orthodontists [48-51]. In addition, postgraduate orthodontics programs should integrate digital and AI literacy, enabling orthodontists to critically appraise AI outputs and identify potential biases [52-54]. These strategies highlight the need for more flexible LLL platforms, ensuring that orthodontic professionals remain competent in their practice.

### Study Limitations and Future Recommendations

Although this qualitative study used a robust research design to ensure credibility and validity, several limitations should be acknowledged. Firstly, this study included only orthodontic professionals in Thailand, which means the generalizability of the findings to broader contexts may be limited. As a qualitative study, the findings are context-specific and are not intended to be statistically generalizable. Future quantitative studies could build on these findings by testing the identified themes and relationships in larger samples, thereby enhancing generalizability and applicability. However, purposive sampling was used to recruit orthodontic professionals from diverse practice settings and with varying levels of working experience. In addition, while this cross-sectional qualitative research allowed the researchers to capture the perceptions of orthodontic professionals at a single point in time, further longitudinal research would be helpful to explore how these patterns change throughout their careers, given that LLL activities are dynamic and subject to temporal changes. Finally, the conceptual framework constructed in this research should be further validated through a quantitative study with a robust research design to strengthen its practical application in dental education.

### Conclusions

LLL engagement among orthodontic professionals represents a complex dynamic that cannot be solely explained by individual motivation. While widely recognized as essential for professional competence, learning strategies are undergoing a significant shift from traditional formal training toward flexible, technology-driven methods such as online communities and generative AI. However, LLL engagement depends on how well these learning expectations align with the realities of clinical practice. The main challenge is not a lack of interest or willingness to learn, but rather a mismatch between professional demands and the time, resources, and institutional support available for ongoing learning. While TPB serves as the analytical framework for this study, enabling a critical examination of how attitudes, social influences, and perceived behavioral control shape intentions at the individual level, this research further highlights that institutional contexts and external influences play key roles in determining whether learning intentions are translated into action. The conceptual framework developed in this research integrates individual intention within broader professional and organizational contexts. These findings emphasize the need for multilevel strategies and validate the proposed framework as a tool for supporting LLL beyond individual responsibility, with implications for future research and educational policy.

## Abbreviations

AI: artificial intelligence
LLL: lifelong learning
TPB: theory of planned behavior
